# Costimulation pathway blockade in kidney transplant recipients with de-novo rheumatoid arthritis 

**DOI:** 10.5414/CNCS108875

**Published:** 2017-02-17

**Authors:** Mohamed Sheta, Samy Riad, Udayakumar Deepak, Naim Issa, Mark Birkenbach, Hassan N. Ibrahim, Aleksandra Kukla

**Affiliations:** 1 South Carolina Nephrology/Hypertension, Orangeburg, SC,; 2 Division of Renal Diseases and Hypertension,; 3 Division of Rheumatic and Autoimmune Diseases, University of Minnesota, Minneapolis, MN,; 4 Division of Nephrology and Hypertension, Mayo Clinic, Rochester, MN, and; 5 Department of LaboratoryMedicine and Pathology, University of Minnesota, Minneapolis, MN, USA

**Keywords:** rheumatoid arthritis, membranous nephropathy, kidney transplant, belatacept

## Abstract

The best approach to treatment of de-novo rheumatoid arthritis in solid organ transplant recipients on typical immunosuppression is not well established. The use of biologics targeting specific cell types, cytokines, and immunological pathways has been gaining interest in the treatment of both, auto- and alloimmunity. We present a case of de-novo rheumatoid arthritis in a kidney transplant recipient 10 years post-transplant while receiving cyclosporine, mycophenolate mofetil, and also prednisone. Initial presentation included features of polymyalgia rheumatica and nephrotic range proteinuria. Kidney biopsy showed membranous nephropathy. The patient was initially treated with methotrexate, while mycophenolate mofetil was discontinued. Clinical symptoms improved, but creatinine significantly increased, which led to discontinuation of methotrexate and mycophenolate mofetil was restarted. The kidney function improved, but the patient experienced a flare of rheumatoid arthritis. Costimulatory blocker, abatacept, was initiated and cyclosporine was gradually tapered off. Graft function remained stable for a follow-up period of 7 years. Joint pain, weakness, and stiffness resolved. Follow-up plain film radiographs at 5 years post initial presentation showed no new joint erosions in hands or feet. Costimulatory blockers may broaden the therapeutic choices of transplant recipients with de-novo autoimmune diseases.

## Case report 

A 63-year-old white male, with a history of end-stage renal disease secondary to granulomatosis with polyangiitis (GPA), received a living donor kidney transplant in 1996. His past medical history included hypertension, coronary artery disease, gout, dyslipidemia, and obstructive sleep apnea. He was maintained on cyclosporine, with trough levels ranging from 80 to 100 µg/L, mycophenolate mofetil 500 mg 3 times a day, and prednisone 5 mg daily. In addition, the patient was on amlodipine, metoprolol, allopurinol, and lisinopril. Baseline creatinine was 1.5 – 1.7 mg/dL. There was no family history of rheumatoid arthritis (RA). Kidney biopsy done 3 years post-transplant was unremarkable. 

10 years post-transplant he developed bilateral shoulder pain, subjective muscle weakness, and stiffness with an elevated erythrocyte sedimentation rate (ESR) above 100 mm/hg and was diagnosed with polymyalgia rheumatica (PMR). He was treated with prednisone 60 mg daily, which was gradually decreased to 40 mg daily with symptomatic improvement. Further attempts to decrease steroid dose were unsuccessful, as symptoms of muscle weakness and myalgias recurred. The patient was referred to our institution for further evaluation. Detailed musculoskeletal examination showed symmetrical synovitis over the proximal interphalangeal, metacarpophalangeal joints, and wrists. He had boutonniere and swan neck deformities of both hands and bilateral lower extremities edema. Cardiopulmonary and abdominal examinations were normal. Relevant laboratory results included serum creatinine 1.6 mg/dL, elevated inflammatory markers and rheumatoid factor (Table 1). IgG antibody against cyclic citrullinated peptide was weakly positive at 21 U/mL (< 5 µ/mL). Urine analysis was positive for albumin (300 mg/dL) without blood; protein to creatinine ratio was 6.8 g/g of creatinine. 

Hand X-rays showed medial subluxation of the first metacarpophalangeal joints bilaterally with scattered poorly-defined erosions. Feet X-rays revealed erosions at the tarsometatarsal joints. Based on clinical presentation, laboratory, and radiologic findings, the patient was diagnosed with RA with PMR features. Based on the clinical symptoms and laboratory findings, the patient met the 1987 American College of Rheumatology (ACR) classification criteria and the 2010 ACR classification criteria for the diagnosis of rheumatoid arthritis. The patient denied a family history of autoimmune diseases. 

The patient underwent transplant kidney biopsy for evaluation of proteinuria. Light microscopy showed moderate interstitial fibrosis and tubular atrophy in a pattern which was attributed to cyclosporine exposure accompanied by mild scattered interstitial lymphoplasmacytic infiltrate and scattered tubulitis. Glomeruli demonstrated basement membrane spikes on silver Jones-stained sections, and direct immunofluorescence showed diffuse and global fine granular staining in peripheral capillary loops for IgG (1+) and C3. Electron microscopy showed subepithelial and intramembranous electron-dense deposits consistent with immune complexes, with rare subendothelial and scattered mesangial deposits also identified. In all, biopsy findings were consistent with membranous nephropathy (MN). There was no histological evidence of relapse of GPA. 

The patient underwent thorough work-up for other autoimmune diseases and malignancy considering the strong association of MN with these conditions. Antinuclear antibody, anti-double-stranded deoxyribonucleic acid antibody, complement (C3, C4, and CH-50) levels, antineutrophil cytoplasmic antibodies (P-ANCA, C-ANCA), prostate specific antigen, hepatitis panel, colonoscopy, CT of the chest, abdomen, and pelvis were all unremarkable. 

**Figure 1.**
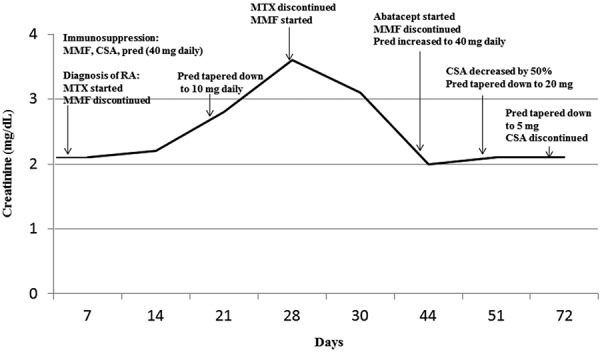
Creatinine and immunosuppressive therapy timeline in the first 72 days post diagnosis of rheumatoid arthritis. MMF = mycophenolate mofetil; CSA = cyclosporine; pred = prednisone; MTX = methotrexate.

The patient was started on methotrexate, while mycophenolate mofetil was discontinued and prednisone dose was tapered down to 10 mg daily. He remained on cyclosporine. After 4 – 6 weeks of therapy, joint pains and stiffness improved, but creatinine increased to 3.6 mg/dL. Repeated kidney transplant biopsy results were consistent with previous findings. Given the lack of alternative explanations for acute worsening of kidney function, methotrexate was discontinued and mycophenolate mofetil was restarted at the dose of 750 mg twice a day. Kidney function improved. Approximately 3 weeks later, he presented with profound fatigue, synovitis over the proximal interphalangeal and metacarpophalangeal joints, and elevated inflammatory markers. Prednisone dose was increased to 40 mg daily and the patient was started on abatacept at the dose of 1,000 mg IV infusion every 4 weeks (weight: 120 kg), together with discontinuation of cyclosporine. 2 weeks after initiation of abatacept, his symptoms of myalgia, fatigue, and arthritis significantly improved. Prednisone was gradually tapered down to the current dose of 5 mg. Renal function stabilized (Figure 1). 7 years post initiation of abatacept, his creatinine is 1.5 mg/dL, and urine protein to creatinine ratio is 0.4 g/g of creatinine, while rheumatoid arthritis continues to be in remission (Table 1). The patient remained on abatacept, 1,000 mg IV infusion every 4 weeks, mycophenolate mofetil 750 mg daily and prednisone 5 mg daily. Plain film radiographs repeated 5 years post original diagnosis showed no new joint erosions in hands or feet. 

## Discussion 

Treatment of RA in kidney transplant recipients poses a significant challenge. Patients are already exposed to immunosuppression and adding additional agents to control RA symptoms may lead to additive toxicity including increased risks of infection and malignancy. The patient described in our case report has not experienced any adverse effects related to immunosuppression. To our knowledge, only a single case report of de-novo RA in a kidney transplant recipient exists in a literature. Forslund et al. [1] reported de-novo seropositive erosive RA in a patient who was 7 years post deceased donor kidney transplantation for end-stage renal disease secondary to diabetes. Similarly to our case, the patient was receiving triple immunosuppression at the time of diagnosis, including cyclosporine, steroids, and azathioprine. Diagnosis was based on the clinical symptoms, elevated RF, CRP, and X-ray examination. The patient was switched from azathioprine to methotrexate, while cyclosporine and prednisone was continued. Contrary to our patient, he tolerated methotrexate well and had a good response to therapy with the resolution of clinical symptoms and normalization of inflammatory markers [1]. In nontransplant population, 30 – 40% of affected individuals may not adequately respond to methotrexate alone. For these patients, biologic disease-modifying antirheumatic drugs (DMARDs) are offered [2]. Our case report proposes that use of co-stimulatory pathway blockade in those challenging patients may be effective in controlling both auto- and alloimmunity. T-cells play a central role in adaptive immunity and therefore are involved in both allogeneic immune responses to transplanted organs and aberrant one, as seen in RA. Two signals are necessary for full T-lymphocyte activation: signal 1 is antigen-specific and is initiated by antigen-binding to the T-cell receptor complex; and signal 2, or costimulatory pathway involving interactions between CD28 molecule on T-lymphocyte surface and its ligands, CD80 and CD86 expressed on antigen-presenting cell (APC). CD86 seems to be more critical in initial T-cell activation, as it is constantly expressed on APC, with rapid up-regulation during initial T-cell-APC interaction. Up-regulation of CD80 requires more prolonged T-cell stimulation, and therefore may be more important in maintaining immune response [3]. 

Cytotoxic T-lymphocyte-associated antigen-4 (CTLA-4) is a transmembrane protein expressed in T-cells after activation that binds both CD80 and CD86 with higher affinity than CD28. By effectively competing with CD28 for ligand binding, CTLA-4 acts to suppress T-cell activation. This property has been exploited to develop immunosuppressive therapeutic agents. The first therapeutic inhibitor of the CD28 pathway comprised the extracellular domain of CTLA-4 fused to the Fc portion of immunoglobulin G (CTLA-4-Ig). This agent significantly prolonged graft survival in rodent transplantation models. Unfortunately, those results were not reproduced in non-human primates, likely due to significantly more potent ability of CTLA-4-Ig to inhibit CD 80 driven co-stimulation as compared to CD 86 [4]. When tested in patients with RA, CTLA-4-Ig use led to significant improvement in signs and symptoms of disease, as well as decreased progression of structural joint damage [5, 6, 7]. The fusion protein CTLA-4-Ig is commercially available as Orencia (abatacept). Subsequently, a modified version of CTLA-4-Ig (belatacept) was developed with much higher avidity to CD86 [3]. This agent is currently approved for maintenance immunosuppression in kidney transplant recipients, and it has shown to be as effective as traditionally used cyclosporine in prevention of acute rejection [8]. When tested in patients with RA in the pilot study, its effectiveness to reduce the signs and symptoms of disease was comparable to abatacept [9]. 

The significant improvement in RA symptoms in our patient can be attributed to the use of abatacept. Its role in maintenance post-transplant immunosuppression, however, is less certain. Low immunologic-risk transplant recipients may be adequately immunosuppressed with mycophenolate mofetil and prednisone alone following cyclosporine withdrawal [10]. Therefore, it seemed feasible to discontinue cyclosporine with an introduction of abatacept in this case. Belatacept could have been considered in our patient, but was not approved in kidney transplantation at his initial presentation. 

## Conclusions 

Abatacept may broaden the therapeutic choices of transplant recipients with de-novo RA and possibly others. This case illustrates its potential utility. 

## Conflict of interest 

Authors declare no conflicts of interest and no sources of support. 

**Table 1.** Laboratory data at the initial presentation and last follow-up.

Investigation	Result at initial presentation	Result at last follow-up
Creatinine (0.6 – 1.25 mg/dL)	1.5	1.5
Albumin (3.4 – 5.0 g/dL)	3.7	4.3
ESR (0 – 20 mm/h)	107	12
CRP (0 – 8 mg/L)	141	
RF (0 – 14 IU/mL)	264	24
Cholesterol (0 – 200 mg/dL)	269	163
Urine protein to creatinine ratio (0 – 0.2 g/g creatinine)	6.8	0.6
